# Isoflurane promotes proliferation of squamous cervical cancer cells through mTOR-histone deacetylase 6 pathway

**DOI:** 10.1007/s11010-020-03884-7

**Published:** 2020-08-24

**Authors:** Wenwen Zhang, Fang Xue, Shangdan Xie, Cheng Chen, Jingwei Li, Xueqiong Zhu

**Affiliations:** Department of Obstetrics and Gynecology, The Second Affiliated Hospital of Wenzhou Medical University, No. 109 Xueyuan Xi Road, Wenzhou, 325027 Zhejiang China

**Keywords:** Isoflurane, Proliferation, Histone deacetylase 6, Cervical cancer, mTOR

## Abstract

This study investigated the effect of isoflurane on the proliferation of squamous cervical cancer cells, with focus on histone deacetylase 6 that is closely related to carcinogenesis. Squamous cervical cancer cells SiHa and Caski were exposed to 1%, 2%, or 3% isoflurane for 2 h, respectively. Cell proliferation was measured with the cell counting kit (CCK-8) assay and determined by BrdU assay. Expression of histone deacetylase 6, phospho-AKT, phospho-mTOR, and proliferating cell nuclear antigen (PCNA) was assessed by Western blot. In order to block the histone deacetylase 6 (HDAC6) expression, siRNA transfection was performed. Isoflurane significantly promoted the proliferation of both SiHa and Caski cells, accompanied by upregulation of PCNA protein expression. Isoflurane increased the level of histone deacetylase 6 protein expression in both cells, and knockdown of histone deacetylase 6 attenuated the pro-proliferation effects of isoflurane. Additionally, activation of AKT/mTOR was found after isoflurane treatment, and mTOR inhibition abolished isoflurane-induced histone deacetylase 6 expression. However, inhibition of AKT phosphorylation had no effect on the expression of histone deacetylase 6 mediated by isoflurane. In conclusion, Isoflurane enhanced proliferation of cervical cancer cells through upregulation of histone deacetylase 6, which was associated with mTOR-dependent pathway, but not AKT-mediated pathway.

## Introduction

Cervical cancer is the fourth most common cancer in women globally with 570,000 cases and 311,000 deaths estimated to occur in 2018, especially squamous cancer [1]. In spite of improvements in surgical techniques and radiation therapy for the management of cervical cancer, the prognosis remains poor. Emerging evidence has shown that perioperative factors including anesthesia technique and anesthetics may result in recurrence of cancer [2]. Limited data have demonstrated that the direct influence of anesthetics, especially inhalational ones on growth of tumor cells is contradictory [3–5]. A volatile anesthetic isoflurane is widely used for patients undergoing cervical cancer surgery. It was documented that isoflurane exerted anti-proliferative activity in pancreatic carcinoma, larynx cancer, and colon cancer cells in a time-dependent manner [3]. In contrast, it was found that isoflurane promoted the growth of renal cancer cells via hypoxia-inducible factor pathway [4]. Increased proliferation of glioblastoma stem cells U251-GSCs was shown under treatment of isoflurane [5]. However, the effect of isoflurane on the growth of squamous cervical cancer cells remains unclear.

Histone deacetylase 6 (HDAC6), originally known as a member of class IIb histone deacetylases family, implicated in multiple cellular processes related to cancer including carcinogenesis, tumor formation, cell adhesion, oncogenic transformation, motility, DNA damage response, cell survival, chaperone function, tumor aggressiveness, stress response, and anchorage-independent proliferation [6, 7]. Four classes of HDACs have been reported, including class I (HDAC1, HDAC2, HDAC3, and HDAC8), class II (class IIa: HDAC4, HDAC5, HDAC7, and HDAC9; class IIb: HDAC6 and HDAC10), class III (sirtuin family of enzymes), and class IV (HDAC11) [8]. Previous studies showed that HDAC6 expression was elevated in many cancers including cervical cancer [9, 10]. Knockdown of HDAC6 was demonstrated to hinder proliferation of cervical cancer HeLa cells, indicating that HDAC6 plays a key role in the carcinogenesis of cervical cancer [10]. PI3K/AKT pathway was revealed to be involved in induction of HDAC6 expression [11]. Isoflurane was reported to upregulate the level of HDAC2 and HDAC3, inducing neuronal apoptosis in vivo [12]. However, whether HDAC6 is involved in the isoflurane-induced proliferation of cervical cancer cells and the related mechanisms are yet to be elucidated.

The present study provides insights into the potential effect of isoflurane on the proliferation of squamous cervical cancer Caski and SiHa cells and the underlying mechanisms. It is also designed to investigate the role of the isoflurane in the regulation of HDAC6 expression in cervical cancer cells and related pathway. It is hypothesized that isoflurane will activate the HDAC6 pathway, thereby promoting cervical carcinogenesis.

## Materials and methods

### Cell culture

Human cervical cancer SiHa and Caski cells were purchased from American Type Culture Collection (ATCC, Manassas, VA, USA). Caski was grown in RPMI-1640 (Gibco, Invitrogen, USA) supplemented with 10% fetal bovine serum (FBS) (Gibco; Invitrogen, USA). SiHa was maintained in Dulbecco’s modified Eagle’s medium (DMEM) (Gibco, Invitrogen, USA) containing 10% FBS. The cells were incubated in a humidified atmosphere containing 5% CO_2_ at 37 °C for growth.

### Isoflurane treatment

SiHa and Caski cells were maintained in a purposely built airtight gas chamber with inlet and exhaust connectors. Isoflurane was delivered through a vaporizer (isoflurane; Drager Vapor 2000, Soma Technology, Bloomfield, USA) into the chamber with 5% CO_2_ /95% air at a rate of 2 L/min for a maximum of 5 min until the desired gas concentration were reached. Gas dose in the chamber were measured at the exhaust part of chamber by using an anesthetic gas analyzer (Datex-Ohmeda, Stirling, UK). The chamber was then moved into a 37 °C incubator (Thermo, USA). The isoflurane group was treated with 1%, 2% or 3% isoflurane mixed with 5% CO_2_ balanced with air for 2 h, respectively. The control one was only exposed to 5% CO_2_/95% air at 2 L/min for 2 h. The following experiments were performed at 24 h post-gas exposure.

### Cell proliferation assay

Cells were cultured in 96-well plates (1 × 10^4^ cells/well) for 24 h and treated with or without isoflurane. We have performed cell viability assay at different time points (0, 6, 12, 18, and 24 h) after 2% isoflurane exposure for 2 h in the pre-experiment and found that isoflurane enhanced cell proliferation with a marked effect at 24 h post-gas exposure. Thus, we perform concentration gradient experiment at 24 h post-gas exposure. After different dose of isoflurane exposure for 2 h, cells were maintained in CO_2_ incubator for additional 24 h. Cell counting kit (CCK-8, Dojindo, Japan) was used to evaluate the viability of cervical cancer cells. Ten μL of CCK-8 was added into each well, and absorbance was measured at 450 nm with a microplate reader. Cell viability was calculated according to the following formula: % viability = (OD450 of test well—OD450 of blank well)/(OD450 of control well—OD450 of blank well) × 100%.

### BrdU assay for cell proliferation

Cells were seeded in 24-well plates (2 × 10^4^ cells/well) for 24 h and treated with or without isoflurane. Cells were treated with BrdU at a final concentration of 30 μg/mL and incubated for 12 h. Subsequently, cells were incubated with 3% H_2_O_2_ for 10 min and fixed with 4% paraformaldehyde for 20 min. After incubated with 2 mol/L HCl in 37 °C for 5 min, cells were permeabilized for 20 min and then blocked in 3% BSA for 30 min in room temperature. The cells were incubated with BrdU monoclonal antibody (1:200 dilution; #5292, Cell Signaling Technology, USA) overnight at 4 °C, followed by second anti-rabbit antibody, incubated at room temperature for 1 h. The cells were stained with diaminobenzidine and hematoxylin for a probable time, observed under a light microscope with 400 × magnification.

### Western blot analysis

The cells were lysed in radio-immunoprecipitation assay (RIPA) buffer. Protein samples were separated insodium dodecyl sulfate–polyacrylamide gel electrophoresis, thereafter transferred into polyvinylidenefluoride (PVDF) membrane. The membranes were probed with the primary antibodies overnight at 4 °C, followed by anti-mouse or anti-rabbit horseradish peroxidase-conjugated secondary antibody (1:1000) for 1 h. Protein bands were visualized by using enhanced chemiluminescence assay kit and quantified using Image J software.The following primary antibodies were used in present work: rabbit anti-HDAC6 (1:1000) (#7558), rabbit anti-phospho-AKT (1:1000) (#4058), rabbit anti-AKT pan (1:1000) (#4685), rabbit anti-phospho-mTOR (1:1000) (#2971), rabbit anti-mTOR (1:1000) (#2983), mouse anti-PCNA (1:1000) (#2586), mouse anti-GAPDH (1:10,000) (#97,166), and rabbit anti-Tubulin (1:1000) (#2146) (Cell Signaling Technology, Denvers, MA). The phospho-AKT antibody we used is phospho-Akt (Ser473) which could recognize endogenous AKT1, 2, and 3 only after phosphorylation at ser473 site. The relative intensity of phospho-AKT was calculated by phospho-AKT versus total AKT and so was the relative intensity of phospho-mTOR. The relative intensity of HDAC6, AKT, mTOR, and PCNA protein was calculated by corresponding protein versus internal control such as GAPDH or ß-Tubulin, respectively.

### siRNA transfection

HDAC6 and scramble siRNAs were purchased from Qiagen (Germany). The HDAC6 siRNA sequences were as follows: 5′-CUUCGAAGCGAAAUAUUAATT-3′. siRNA transfection reactions were performed using Lipofectamine RNAi Max (Invitrogen, USA) in accordance with the manufacturers' instructions. Twenty-four hours after transfection, the cells were treated with isoflurane and measured by proliferation assay and Western blot analysis at 24 h post-gas exposure.

### AKT inhibitor and mTOR inhibitor/agonist treatment

SiHa and Caski cells were cultured in six-well plates at a density of 7 × 10^5^ cells per well. AKT inhibitor LY294002 20 μM (Cell Signaling Technology, USA), which could significantly block phosphorylation of Akt1, 2, and 3, was used to treat cells for 1 h prior to isoflurane exposure. The cells were pretreated with mTOR inhibitor rapamycin 10 μM (Cell Signaling Technology, USA) for 2 h, followed by isoflurane exposure. MHY1485 10 μM (MedChemExpress, USA), mTOR agonist, was used to treat cells with isoflurane exposure.

### Statistical analysis

Statistical analysis of the data, expressed as means ± standard deviation, were performed by one-way analysis of variance (ANOVA) followed by Dunnett’s T3 test (when data were only compared to the controls) and Newman-Keuls post hoc comparison (when data were compared between all groups). Unpaired t test was used to compare two independent samples. Statistical Product and Service Solutions (SPSS) 17.0 statistical software was used for the statistical analysis. A two-sided *P* value of less than 0.05 was considered statistically significant.

## Results

### Isoflurane promotes proliferation of cervical cancer cells

**Fig. 1:**
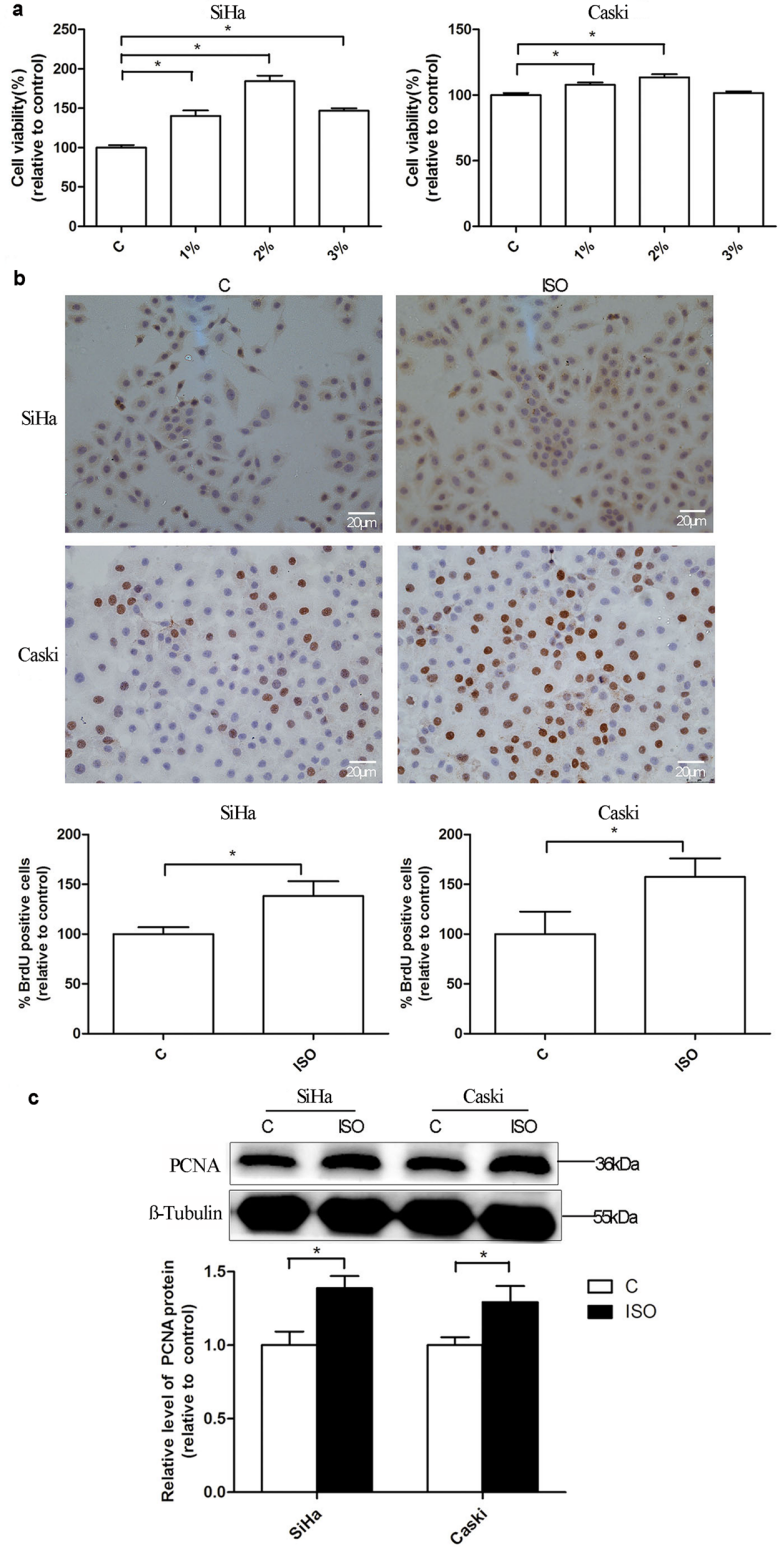
Cell proliferation of cervical cancer cells after isoflurane treatment. Caski and SiHa cells were treated with or without different concentration (1%, 2%, and 3%) of isoflurane in 5% CO_2_ balanced with air for 2 h, and cell viability assay with CCK-8 assay (**a**) was performed at 24 h post-gas exposure. BrdU assay was evaluated at 24 h post-2% isoflurane exposure for 2 h (SP staining × 400) (**b**). Western blot analysis of PCNA was performed at 24 h after 2% isoflurane exposure for 2 h (**c**). Data were shown as mean ± standard deviation from three independent experiments. One-way ANOVA with Dunnett’s T3 test (**a**); Unpaired t test (**b** and **c**); *P < 0.05. *C* control, *ISO* isoflurane

Cervical cancer cells SiHa and Caski were exposed to different concentrations of isoflurane for 2 h. Then CCK-8 assay and BrdU assay were performed to determine the cell viability and cell proliferation at 24 h post-gas exposure. Western blot was also used to evaluate the proliferation-related protein in cervical cancer cells. When compared to the control group, increased viability of SiHa cells was observed at 24 h post-gas exposure with 1%, 2%, or 3% isoflurane, with the highest level after 2% isoflurane treatment (Fig. 1a). Isoflurane also enhanced viability of Caski cells with significant increases at 24 h post-gas exposure with 1% and 2% isoflurane as compared to the control ones. No significant effect of isoflurane was shown on Caski cells at 3% isoflurane. BrdU assay also indicated that isoflurane enhanced proliferation of both cells (Fig. 1b). Additionally, data revealed that PCNA expression in both cells was also markedly elevated in 2% isoflurane-treated cells, as compared to the control ones (Fig. 1c).

### Isoflurane increases HDAC6 expression in cervical cancer cells

**Fig. 2:**
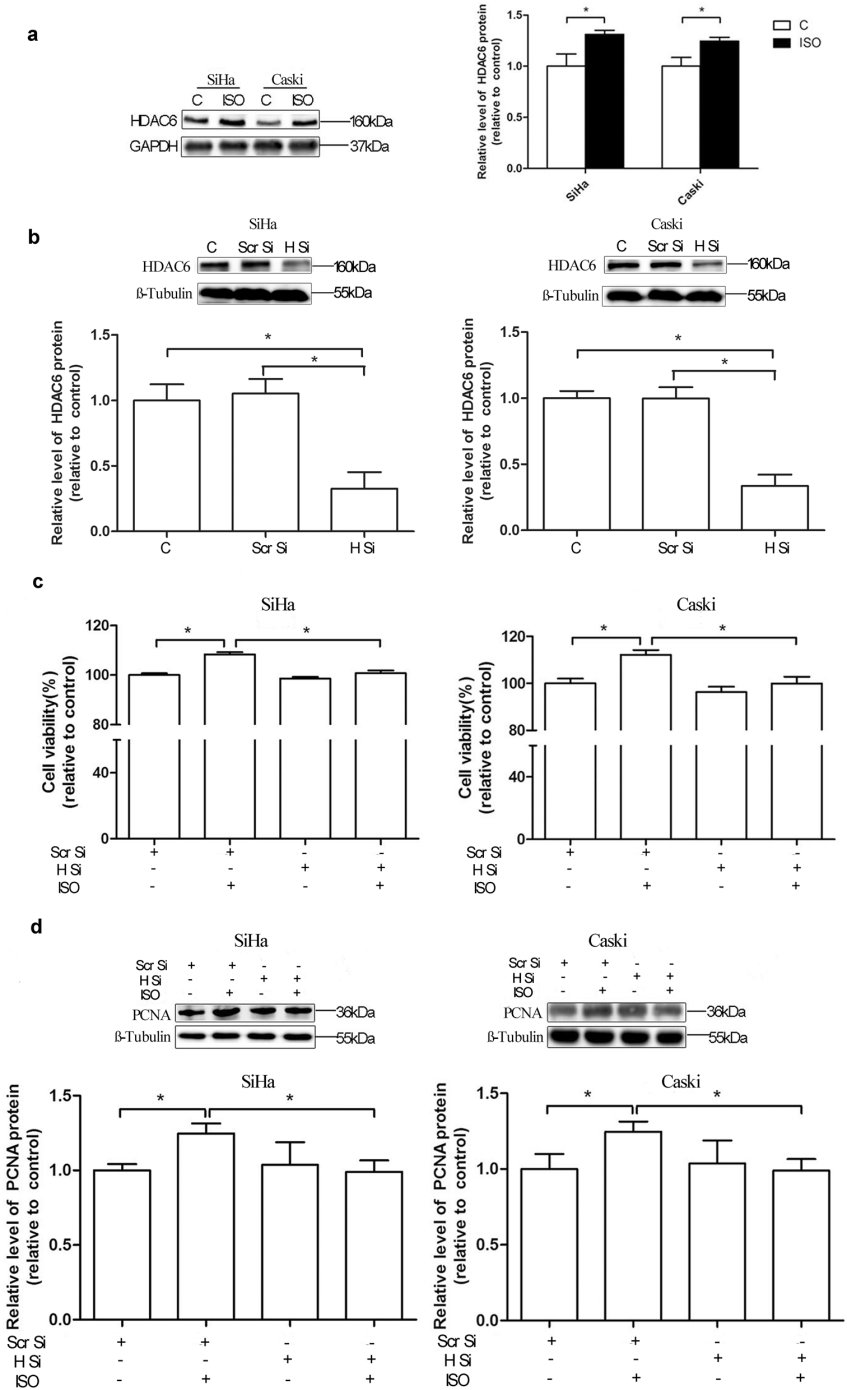
Effects of HDAC6 on isoflurane-induced proliferation of both SiHa and Caski cells. Cells were treated with or without 2% isoflurane and evaluated with Western blot analysis of HDAC6 (**a**) 24 h post-gas exposure. The expression of HDAC6 protein in cervical cancer cells transfected with HDAC6 small interfering RNA (siRNA) was evaluated with Western blot analysis (**b**). After transfected with HDAC6 siRNA, the cells were exposed to 2% isoflurane for 2 h. Then the cell proliferation was detected by CCK-8 assay (**c**). Western blot analysis of PCNA (**d**) was performed 24 h post-gas exposure for proliferation of SiHa and Caski cells. Data were presented as mean ± standard deviation from three independent experiments. One-way ANOVA with Newman–Keuls corrections (**b**, **c** and **d**); Unpaired t test (**a**); *P < 0.05. *C* control, *H Si* HDAC6 siRNA, *ISO* isoflurane, *Scr Si* scramble siRNA

To explore the effect of isoflurane treatment on HDAC6 protein expression in cervical cancer cells, SiHa and Caski cells were exposed to 2% isoflurane for 2 h and then harvested for Western blot at 24 h post-gas exposure. The expression of HDAC6 protein in SiHa and Caski cells after isoflurane treatment was dramatically increased in comparison with control one (*P* < 0.05) (Fig. 2a). In order to elucidate the correlation between expression of HDAC6 and the role of isoflurane on cervical cancer cells, further study was performed to investigate the knockdown effect of HDAC6 on the proliferative activity of SiHa and Caski cells. When compared to 2% isoflurane only group, cell viability of both cells was markedly decreased in group treated with knockdown of HDAC6 and isoflurane (Fig. 2b, c). In addition, knockdown of HDAC6 combined with isoflurane group was also found to reduce expression of PCNA in both cells, compared with 2% isoflurane only group (Fig. 2d).

### Isoflurane increases HDAC6 expression not via AKT pathway

**Fig. 3:**
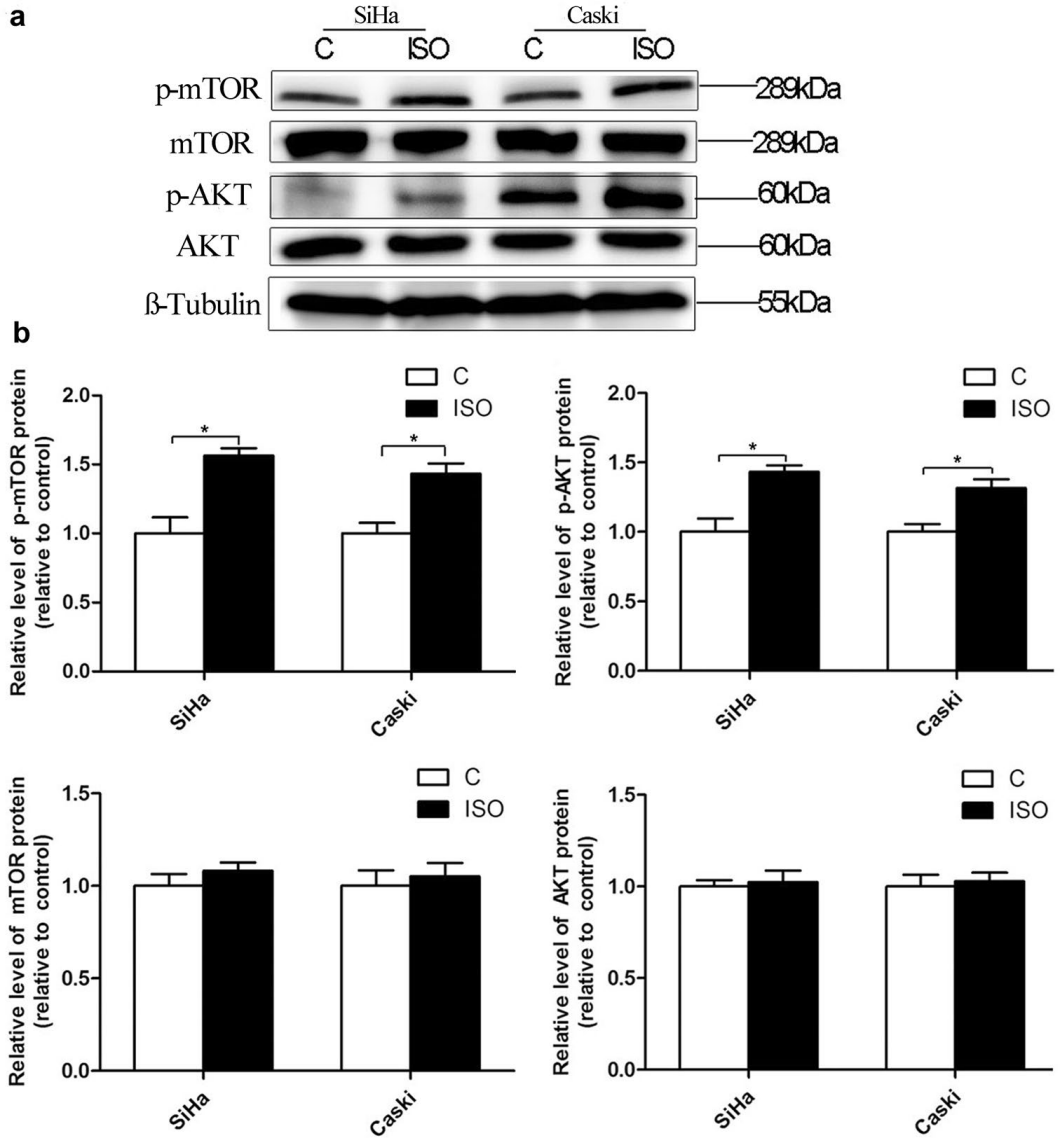
Isoflurane activates AKT-mTOR pathway in cervical cancer cells. Caski and SiHa cells were treated with or without 2% isoflurane for 2 h, and upstream HDAC6 effector phosphorylated-AKT (p-AKT), total AKT, phosphorylated-mTOR (p-mTOR), and total mTOR were analyzed by Western blot at 24 h post-gas exposure (**a** and **b**). Data were presented as mean ± standard deviation from three independent experiments. Unpaired t test; *P < 0.05. *C* control, *ISO* isoflurane

**Fig. 4:**
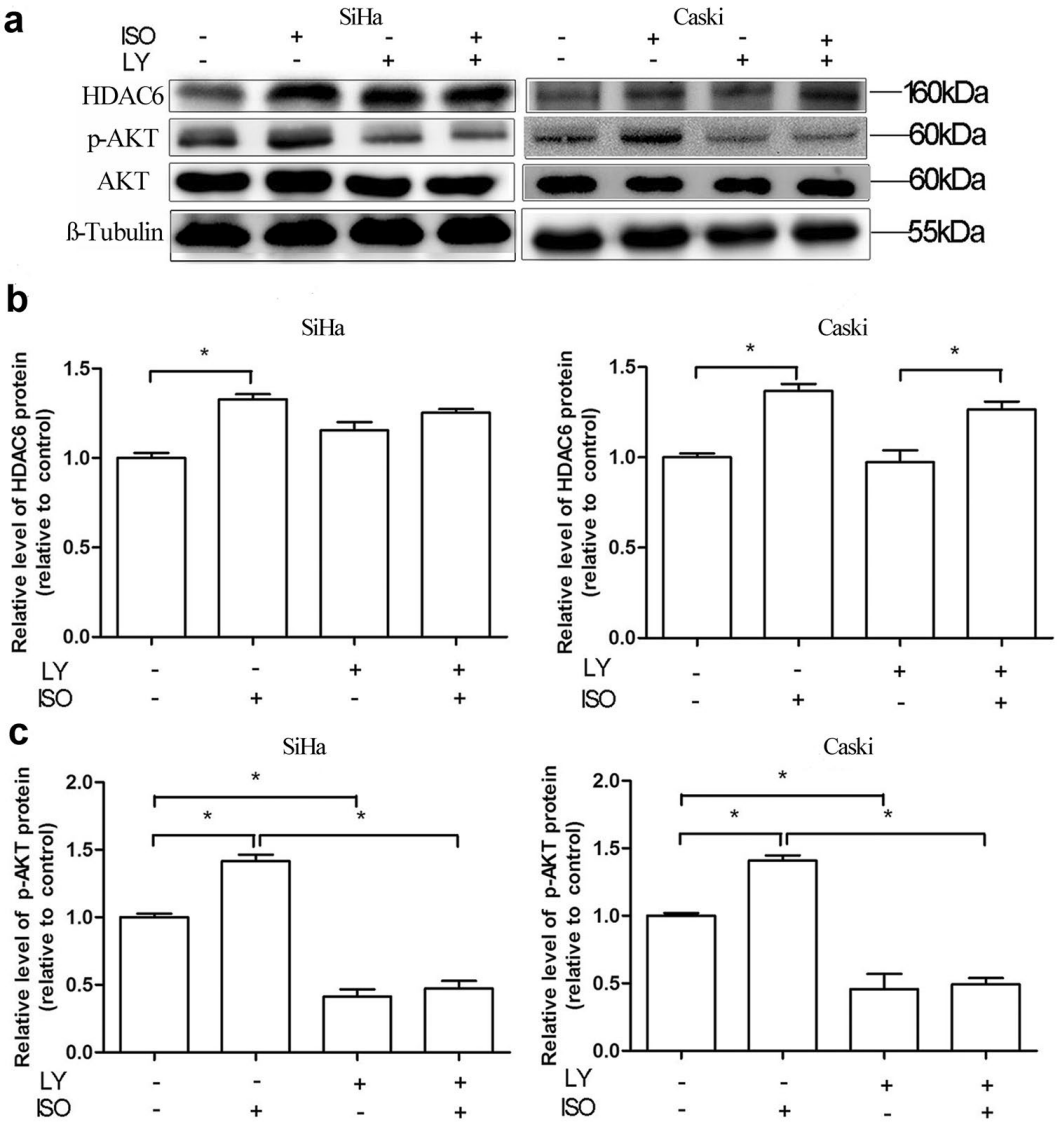
Isoflurane increases HDAC6 not via AKT pathway. SiHa and Caski cells were pretreated with an AKT inhibitor (LY294002) prior to treatment with 2% isoflurane for 2 h, followed by Western blot to detect changes of HDAC6 and p-AKT expression (**a**). Data were presented as mean ± standard deviation from three independent experiments. One-way ANOVA with Newman–Keuls corrections (**b** and **c**); *P < 0.05. *C* control, *ISO* isoflurane, *LY* LY294002

To investigate the possible mechanism that responsible for the upregulation of HDAC6 caused by isoflurane, the effects of AKT on HDAC6 expression were assessed following treatment with AKT-specific inhibitor. We detected that expression of p-AKT protein was significantly increased in both cells after isoflurane exposure (*P* < 0.05) (Fig. 3a, b). However, there was no effect of isoflurane on the expression levels of total AKT (*P* > 0.05) (Fig. 3a, b). LY294002 alone (AKT inhibitor) group was found to upregulate HDAC6 (Fig. 4a, b) in SiHa cells. However, this LY294002-mediated activation of HDAC6 was not noted using Caski cells. The expression of p-AKT was markedly down-regulated in the group that treated with both AKT inhibitor (LY294002) and isoflurane, as compared to isoflurane only group (Fig. 4a, c). However, treatment with LY294002 and isoflurane did not reduce the expression of HDAC6 in both cells in comparison with cells that treated only with isoflurane (Fig. 4a, b). These findings indicate that AKT pathway is not associated with isoflurane-induced HDAC6 expression.

### Isoflurane increases HDAC6 expression through mTOR pathway

**Fig. 5:**
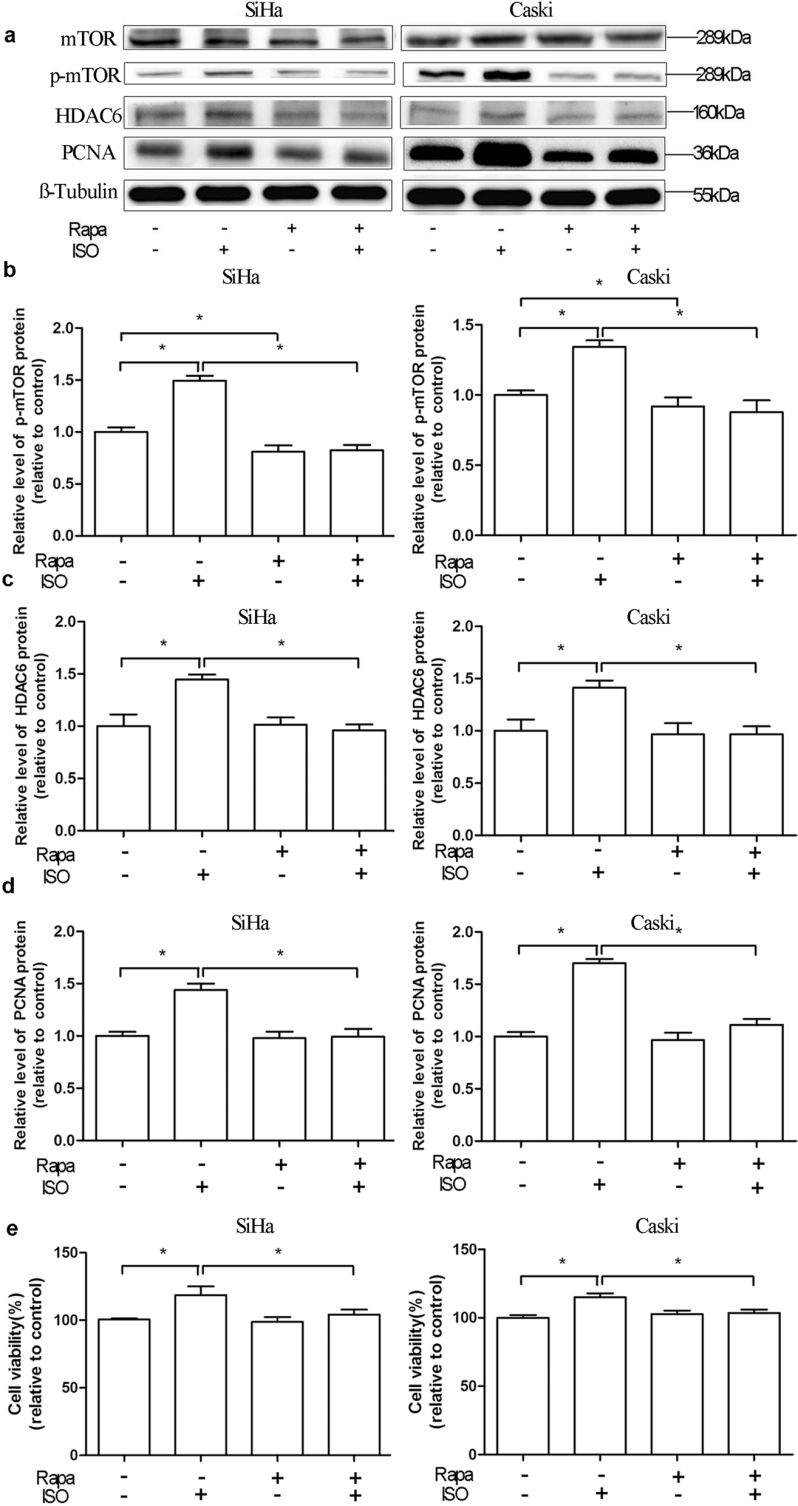
Isoflurane increases HDAC6 via mTOR pathway. SiHa and Caski cells were pretreated with an mTOR inhibitor (Rapa) prior to treatment with 2% isoflurane for 2 h, followed by Western blot to detect changes of p-mTOR, HDAC6, and PCNA expression (**a**, **b**, **c** and **d**) and CCK-8 assay for proliferation (**e**). Data were presented as mean ± standard deviation from three independent experiments. One-way ANOVA with Newman-Keuls corrections (**b**, **c**, **d** and **e**); *P < 0.05. *ISO* isoflurane, *Rapa* rapamycin

**Fig. 6:**
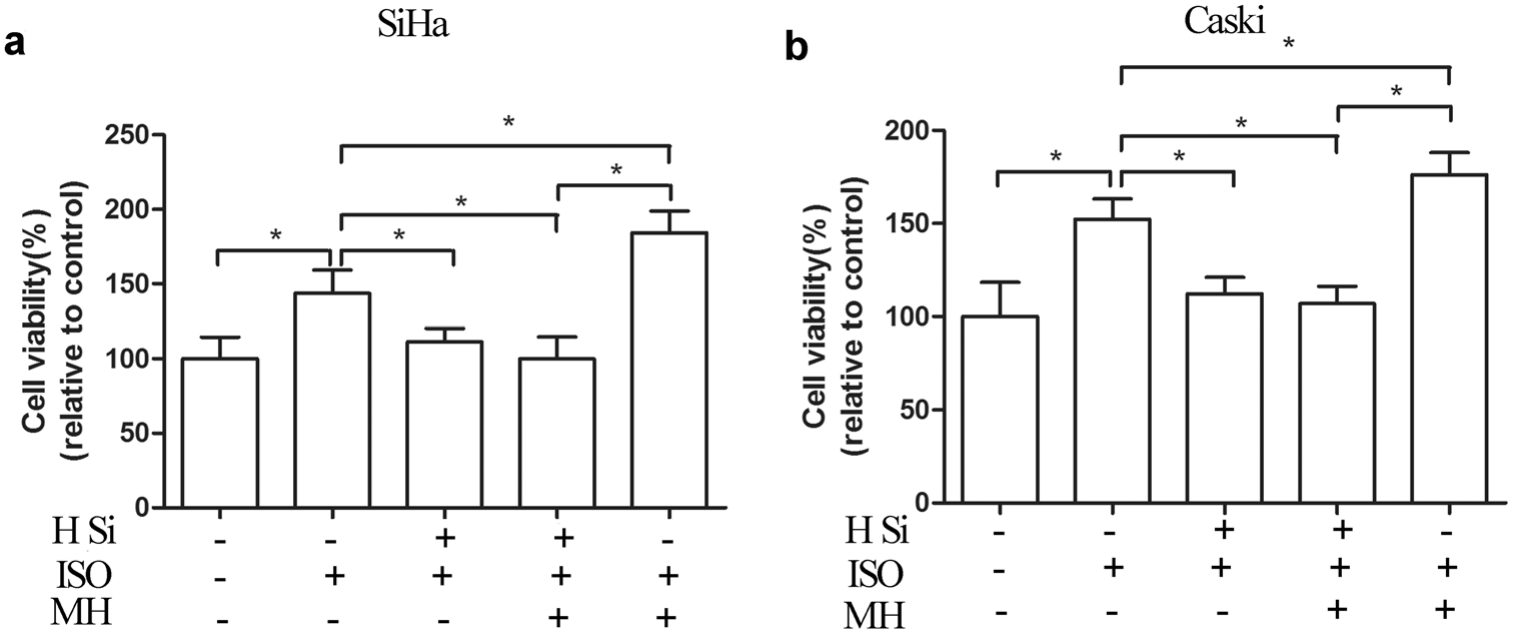
Effects of mTOR-HDAC6 on isoflurane-induced proliferation of both SiHa and Caski cells. SiHa and Caski cells were pretreated with HDAC6 siRNA prior to treatment with 2% isoflurane and mTOR agonist MHY1485 for 2 h, followed by CCK-8 assay for proliferation (**a** and **b**). Data were presented as mean ± standard deviation from three independent experiments. One-way ANOVA with Newman–Keuls corrections; *P < 0.05. *H Si* HDAC6 siRNA, *ISO* isoflurane, *MH* MHY1485

To explore whether mTOR participates in upregulation of HDAC6 expression induced by isoflurane, we found that p-mTOR protein expression was increased in both cells after the treatment with isoflurane (*P* < 0.05) (Fig. 3a, b). However, no difference of total mTOR expression was found in both cells after isoflurane exposure (*P* > 0.05) (Fig. 3a, b). Reduced expression of p-mTOR was detected in the group that exposed to both mTOR inhibitor (rapamycin) and isoflurane when compared to isoflurane only group (Fig. 5a, b). When compared to isoflurane only group, HDAC6 and p-mTOR levels were markedly decreased in group receiving both mTOR inhibitor rapamycin and isoflurane (Fig. 5a–c). The expression of PCNA was significantly reduced in the group receiving both rapamycin and isoflurane, as compared to isoflurane only group (Fig. 5a, d). Rapamycin also abolished the proliferative effect of isoflurane on Caski and SiHa cells (Fig. 5e). It was found that the proliferation of cell in isoflurane combined with mTOR agonist (MHY1485) group was increased as compared to that in isoflurane only group (Fig. 6). Additionally, Treatment with MHY1485 did not promote proliferation of SiHa and Caski cells in the presence of isoflurane and HDAC6 siRNA. These data suggest that mTOR pathway is related to isoflurane-induced HDAC6 expression, which markedly promotes the proliferation of squamous cervical cancer cells.

## Discussion

**Fig. 7:**
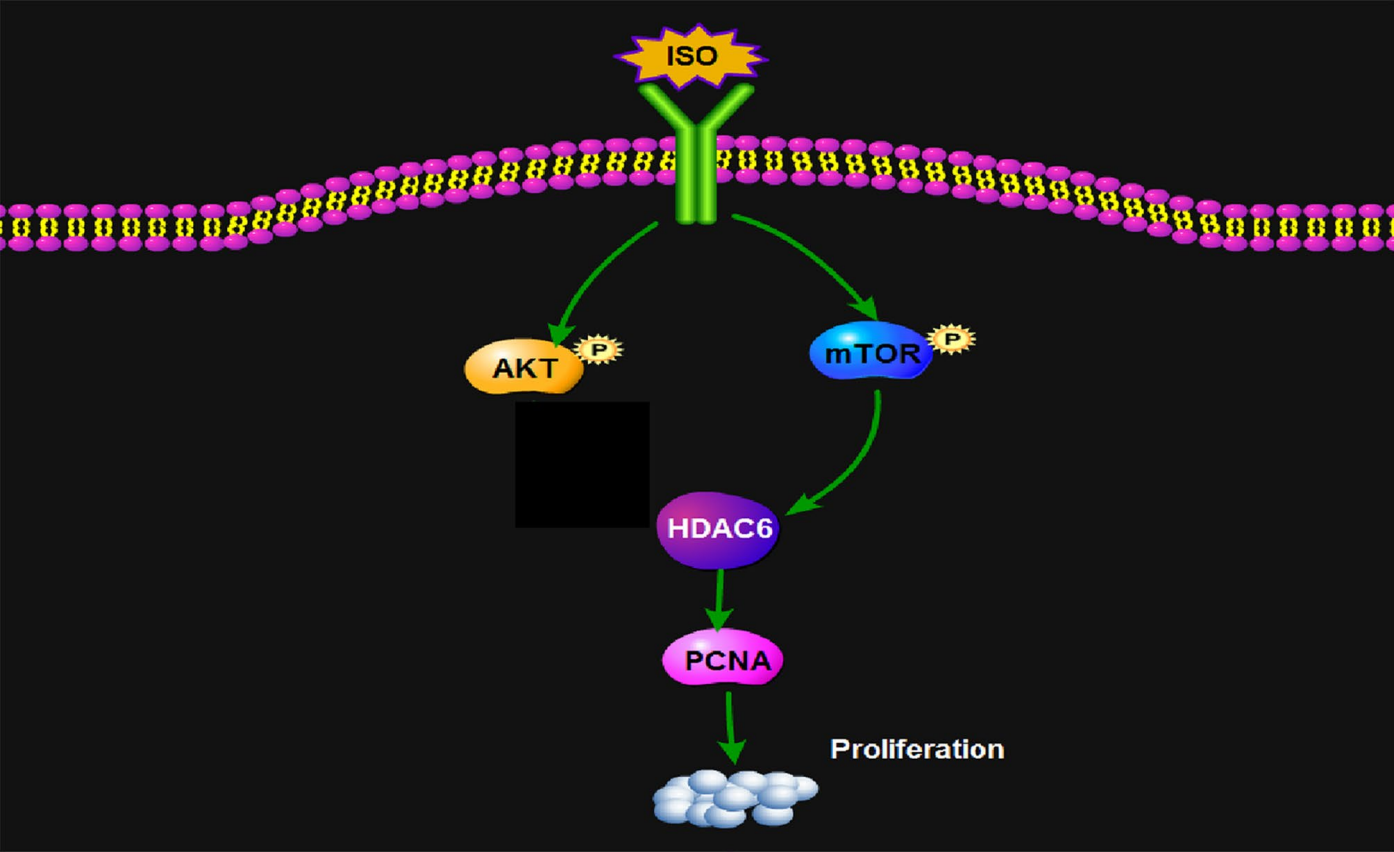
The related mechanism responsible for isoflurane-induced proliferation of squamous cervical cancer cells. Isoflurane increases the expression of HDAC6 via the mTOR pathway. HDAC6 may, thus, be responsible for isoflurane-induced proliferation of squamous cervical cancer cells. Isoflurane may activate AKT pathway, which is not associated with upregulation of HDAC6. *ISO* isoflurane

The present study provides evidence that isoflurane promotes growth of cervical cancer cells, which are mediated by upregulation of HDAC6. In addition, we hypothesized that induction of HDAC6 by isoflurane was mediated by mTOR pathway, which enhanced the proliferation of cervical cancer cells, but not AKT pathway in Fig. 7.

It was well known that one of the characteristic distinguishing tumor cells from normal ones is the abnormal proliferative capacity [4]. The effects of isoflurane on cancer cell proliferation are controversial. Isoflurane was found to markedly suppress the proliferation and viability of human laryngeal papilloma cells via inhibiting activity of cyclooxygenase-2 [13]. However, in the study of non-small cell lung cancer cell lines A549 and H1299, isoflurane was shown to enhance cell proliferation in a dose-dependent manner via activation of the AKT-mTOR signaling pathway [14]. To simulate clinical status of anesthesia in cervical cancer surgery, SiHa and Caski cells were exposed to three commonly used doses of isoflurane (1%, 2%, and 3%) for 2 h. As a thymidine analog, BrdU can infiltrate the transcribed DNA molecule in place of thymine (T) during cell proliferation, which can reflect DNA replication activity and has been a major marker of cell proliferation [15]. The changes of PCNA protein are consistent with DNA synthesis. Detection of PCNA protein expression in cells can be used as an indicator of cell proliferation [16]. We observed that isoflurane promoted the proliferative activity of both cervical cancer cells with increased expression of PCNA and BrdU-positive cells.

HDAC6 is mainly expressed in the cytoplasm, implicated in carcinogenesis, development, and metastasis [17, 18]. Increasing evidence revealed that deubiquitination and ubiquitination of HDAC6 were involved in carcinogenesis [19]. HDAC6 deacetylates survivin and augments levels of survivin in the cytoplasm, blocking programmed cell death by inactivating caspase proteins, thus inducing oncogenesis [20]. Our study showed that isoflurane exposure caused markedly increase of HDAC6 expression in the cervical cancer cells. Knockdown of HDAC6 mitigated the protumorigenic effects of isoflurane on SiHa and Caski cells, supporting this as a mechanism.

Interestingly, the PI3K/AKT signaling pathway is commonly disrupted in cancers, with AKT as a key member of the pathway, playing a prominent role in many processes involved in carcinogenesis [21]. It was demonstrated an association between pretreatment p-AKT level and poor survival outcome in patients with cervical cancer after chemo-radiotherapy [22]. In a study of human breast cancer stem cells, a decrease of HDAC6 protein expression in R2N1d (highly tumorigenic M13SV1R2N1 cell lines from a breast epithelial cell type with stem cell phenotypes cells) cells after treatment with the PI3K inhibitor, suggesting that HDAC6 expression could be regulated by PI3K/AKT activity [11]. It was reported that miR-206 is involved in the progression of human head and neck squamous cell carcinoma by mediating HDAC6 through PTEN/AKT/mTOR pathway [23]. However, in the present study, we observed that isoflurane increased p-AKT and p-mTOR expression in cervical cancer cells. AKT inhibitor-mediated activation of HDAC6 was found in SiHa cells, but not in Caski cells. As the metastatic potential and HPV types of the two cancer cells are different (SiHa: established from situ tissue samples with HPV-16 positive; Caski: established from small intestinal mesenteric metastases with both HPV-16 and -18 positive), it seems that the sensitivity of such two cancer cells to AKT inhibitor-mediated activation of HDAC6 is also different. The effect of isoflurane on HDAC6 expression was abolished by mTOR inhibitor, but not AKT inhibitor. Results suggest that AKT seems not the direct upper stream factor of HDAC6 in isoflurane-induced proliferation of cervical cancer cells. Our findings indicate that activation of mTOR pathway might be an explanation for the isoflurane-induced expression of HDAC6 in cervical cancer cells. Additionally, mTOR agonist had no effect on the proliferation of cervical cancer cells in the presence of isoflurane and HDAC6 siRNA, indicating that isoflurane-induced proliferation of cells is mediated by mTOR-dependent HDAC6 pathway.

The present research has some limitations. Given volatile anesthetics commonly used in combination with other agents such as propofol as general anesthesia and the enormous heterogeneity among individual types of tumor, it is necessary to explore the effect of combination with isoflurane and propofol on different gynecological cancer cell lines including cervical cancer cells in further study. As tumor in vivo model can accurately simulate the tumor microenvironment and the transport of anesthetics to tumor cells, in vivo study is under investigation by our group.

In conclusion, isoflurane enhances proliferation of cervical cancer cells mediated by the activation of HDAC6 via mTOR pathway. Collectively, it provides novel evidence for a direct effect of isoflurane on tumorigenesis of cervical cancer and the underlying mechanism by stimulating of mTOR-HDAC6 pathway.

## Data Availability

All data are fully available.
